# Deceptive Adherence to Anticoagulation in Secondary Stroke Prevention

**DOI:** 10.1155/2022/5318259

**Published:** 2022-07-11

**Authors:** Riina Vibo, Juhan-Mats Kuningas, Prinno Tsakuhhin, Janika Kõrv

**Affiliations:** Department of Neurology and Neurosurgery, University of Tartu, Estonia L. Puusepa 8, 50406 Tartu, Estonia

## Abstract

**Background:**

Oral anticoagulants (OAC) effectively reduce the risk for ischemic stroke in patients with atrial fibrillation (AF). We aimed to assess OAC treatment adherence in secondary stroke prevention and to find predictors of adherence using individualized patient data.

**Methods:**

This retrospective cohort study included patients with a discharge diagnosis of ischemic stroke and AF from Tartu University Hospital from 2017 to 2018. Data from patient charts and the Electronic Hospital Information, Estonian Electronic Prescription, and Estonian Electronic Health Record systems were registered.

**Results:**

Of the 353 patients, 237 (67%) were prescribed OAC treatment at discharge and during the first year after stroke, 202 (85%) of them used OAC treatment. The mean adherence was 81%, while only 68% had good adherence. Reduced non-vitamin K antagonist OAC (NOAC) dose was used in 68 patients (39%), which was justified in 23 (34%). First-ever stroke occurrence was the only significant factor for good treatment adherence in logistic regression analysis. There were 47 patients (23%) with complications among the patients on OAC treatment. Majority of the patients (70%) with hemorrhagic complications and 52% of patients with thromboembolic complications had good treatment adherence

**Conclusions:**

Our study showed that OAC treatment adherence following stroke was modest and first-ever stroke was the only predictor of good or full treatment adherence.

## 1. Background

Oral anticoagulants (OAC) effectively reduce the risk for ischemic stroke in patients with atrial fibrillation (AF) [1]. There are several guidelines on how to introduce OAC in clinical practice [2]. Despite the proven effectiveness of OAC in patients with AF, this treatment is still underused both in primary stroke prevention and secondary stroke prevention [3, 4].

Poor adherence to treatment, including OACs, is a worldwide problem when it comes to chronic diseases [5]. However, OACs in patients with AF and stroke provide significant risk reduction [1] and thus, adherence to OACs in these patients is of the utmost importance. Moreover, in our opinion, there is a positive association between good adherence and treatment effectiveness.

While there have been numerous studies reporting real-world data on OAC use and therapy adherence in AF patients, much less is known about OAC treatment following an ischemic stroke [6–14]. These studies on OAC in stroke patients have reported somewhat different results depending on the aim of the study, methods used, and type of patient cohort and/or time of evaluation after stroke. Therefore, more information on factors associated with treatment adherence following stroke occurrence is needed. The proportion of cardioembolic stroke in previous studies from our country, Estonia, has been higher compared to that of other countries [15, 16]. Therefore, the adherence of patients with AF to OAC treatment is of high priority in stroke prevention. We determined to assess treatment adherence to OACs in the secondary prevention of stroke and to find predictors of adherence using a retrospective analysis of individualized patient data.

## 2. Methods

### 2.1. Data Sources and Study Population

We included patients hospitalized in the Department of Neurology at Tartu University Hospital with a discharge diagnosis of ischemic stroke and AF from January 1, 2017, to June 30, 2018. All of the patients were managed by the stroke neurologist in the stroke unit. The follow-up period was 1 year from the diagnosis of index stroke.

Data were obtained from four databases: (a) the patients' charts from Tartu University Hospital, (b) Electronic Hospital Information system, (c) Estonian Electronic Prescription (EP) system, and (d) Estonian Electronic Health Record (EHR) system. Individual patient data from the aforementioned sources were reviewed and analyzed in detail.

Based on the medical charts, we registered the patients' age, sex, renal function (estimated glomerular filtration rate, eGFR), cardioembolic (CHA_2_DS_2_-VASc) [17] and bleeding risk (HAS-BLED) [18], stroke severity (National Institutes of Health Stroke Scale, NIHSS) on admission and at discharge, history of OAC treatment before the index stroke, and recommendations for anticoagulation therapy during hospitalization.

Data on all prescriptions issued in Estonia are recorded in the electronic EP and are linked to individual patients' medical documents. This system provides data on both available prescriptions and the medication purchases of each individual. The following information was obtained for each OAC prescription for enrolled patients during the follow-up period: the name of the medication (dabigatran, apixaban, rivaroxaban, or warfarin), the date the medication was dispensed, the number of tablets dispensed, and the tablet formulation.

The EHR is a unique system that integrates data from all healthcare providers nationwide (including general practitioners) to create a common record of all medical information for each individual. All of the treatment complications during the follow-up period were registered. The complications included clinically relevant hemorrhages (intracranial, gastrointestinal, and other kinds of hemorrhages), thromboembolic complications (systemic embolic events, stroke transient ischemic attack), or death. If the patient died at home, the cause of death could not be obtained from this database.

### 2.2. OAC Treatment Adherence

Adherence to OAC therapy was defined as the patient's medication use as prescribed during the study period. For each patient, we used the medication possession ratio (MPR) using the prescribed individual daily dose for each patient to describe 1-year treatment adherence [19]. We measured adherence based on individual purchased prescriptions in EP, assuming the provided medications were taken. Early/late refill information was not available and thus not accounted for in the calculations. If a patient discontinued treatment by or during the follow-up period, the adherence was calculated until the discontinuation. If the patient died during the study, the MPR was calculated using the time the patient was alive. We defined “full adherence” as coverage with sufficient medication intake for the whole period with no breaks in treatment. “Good adherence” referred to sufficient medication that covered 80% to 99% of the days of the specified treatment period and “nonadherence” if the purchased medication covered less than 80% of the period. We defined the date of stroke onset as the index date.

To evaluate the proportion of patients who met the indications for dose reduction, we used GFR, body weight, and age for the calculations. The dosages were assessed according to the recommendations in the summary of product characteristics for each medication. If the prescribed dose was in line with patient characteristics and the summary of product characteristics, it was defined as “correct dose.” Adherence to warfarin was defined according to the individual prescribed dose. Edoxaban was not registered in Estonia during the study period.

All methods are in accordance with relevant guidelines and regulations. The study was approved by the Research Ethics Committee of the University of Tartu.

### 2.3. Statistical Analysis

Data were described using the mean (standard deviation), median (interquartile range), and proportions (absolute and relative frequencies) as appropriate. The chi-square test was used to compare the distributions of categorical variables, and the *t*-test, Wilcoxon rank-sum test, one-way analyses of variance, or Kruskal-Wallis test were used for numerical variables.

Variables included in the multiple logistic regression model as potential predictors of good/full adherence (MPR ≥ 80%) as opposed to nonadherence were selected a priori and included age, sex, stroke recurrence, CHA_2_DS_2_-VASc score, HAS-BLED score, eGFR, NIHSS score at discharge, and OAC medication used. Odd ratios with corresponding 95% confidence intervals were calculated. *p* values < 0.05 were considered statistically significant. Data were analyzed using Stata software (version 14.2; StataCorp, College Station, TX, USA).

## 3. Results

**Figure 1:**
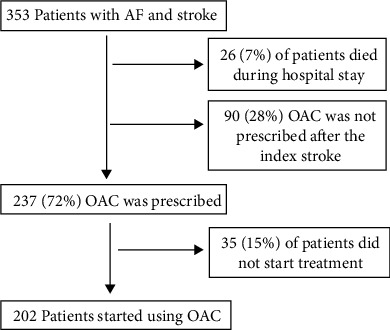
Patient recruitment.

A total of 353 patients with a mean age of 78.8 (±9.5) years were hospitalized during the 18-month study period (Figure 1). The patient demographics are shown in Supplementary Table 1. Women were significantly older and had more occurrences of severe stroke than did men.

Among the patients, 251 (71%) had AF diagnosed before stroke. Of them, 116 (46%) were taking anticoagulants. Prior to the index stroke, 135 (54%) patients were not on OAC treatment: in 34 (25%) patients, the prescribed OAC treatment had been discontinued earlier; for 22 (16%), the reason for not using OAC was documented in the patient files; 18 (14%) refused OAC treatment; and for 61 (45%), the reasons were unknown.

During the hospital stay, 26 (7%) patients died and were therefore excluded from further analysis.

### 3.1. Anticoagulation after Stroke

For 237 patients (72%), OAC treatment was recommended (43%) or started (57%) during their hospitalization. During the mean follow-up of 348 ± 57 (40–365) days, 202 (85%) patients used OAC and were included in further analysis. Of them, 83 (41%) used apixaban; 62 (30%), rivaroxaban; 29 (14.5%), dabigatran; and 28 (14.5%), warfarin. There were 153 (76%) OAC users who experienced a stroke for the first time. If OAC treatment was initiated at the hospital, 96% of the patients continued the treatment at home. However, out of those who were prescribed OAC treatment initiation at home due to moderate/severe stroke, only 65% started the treatment after discharge.

Out of the 202 patients who started using anticoagulants, 21 (10%) died and 15 (8%) discontinued treatment during the follow-up period.

The patients who did not start or were not prescribed OAC after stroke occurrence (*n* = 151) were significantly older (82.5 ± 9.1 years; *p* < 0.001) and had more severe stroke (median NIHSS [IQR] at discharge 12 [5–20]; *p* < 0.001), 82% died within 1 year following the index stroke. The characteristics of patients who were not prescribed OAC and those who did not purchase the prescription were not significantly different.

### 3.2. Treatment Adherence

**Figure 2:**
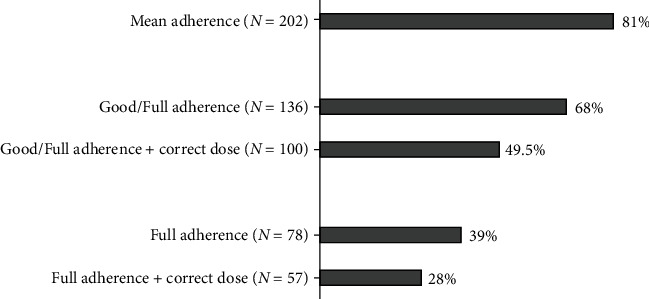
Proportion of patients in each treatment adherence group.

The mean treatment adherence was 81% (MPR, 0.81 ± 0.24). A total of 136 (68%) patients had good or full treatment adherence (treatment available ≥ 80% of the time). Patient characteristics according to adherence are shown in Table 1. Adherence was significantly better in patients with first-ever stroke (*p* = 0.005). The proportion of patients in each treatment adherence group is shown in Figure 2.

For the assessment of factors related to good treatment adherence, a multiple logistic regression model was used. Only first-ever stroke remained a significant factor for good treatment adherence (see Table 2).

Reduced NOAC dosage was prescribed for 68 patients (39%). This was justified only in 23 (34%) patients.

### 3.3. Complications

There were 47 patients (23%) with complications among the patients on OAC treatment. During the 1-year follow-up period, 21 (10%) patients died. The cause of death were complications in 10 (5%) patients (5 gastrointestinal hemorrhages, 2 intracranial hemorrhages, 2 myocardial infarctions, and 1 ischemic stroke). In 11 (5%) patients, the cause of death was unavailable. Among the surviving 181 patients, there were 20 (11%) thromboembolic events and 6 (3%) hemorrhagic events. Recurrent stroke or transient ischemic attack was diagnosed in 16 (8%), myocardial infarction or systemic embolism in 4 (2%), intracranial hemorrhage in 1 (0.5%), gastrointestinal hemorrhage in 3 (1.5%), and other hemorrhagic complications in 2 (1%) patients.

**Figure 3:**
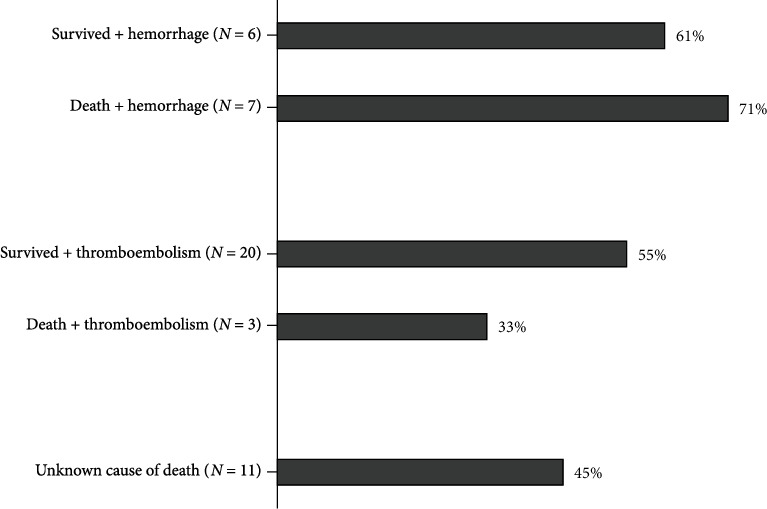
Proportion of patients with good/full treatment adherence among patients with complications (each category shows the number of patients in this group, and the corresponding percentage refers to the proportion of patients with good or full treatment adherence in this group).

The treatment adherence of patients with any complications is shown in Figure 3. The majority of patients with hemorrhagic complications had good or full treatment adherence. Two of the three patients with intracranial hemorrhage were nonadherent (one of them had discontinued OAC 4 months before the complication occurred). Patients with thromboembolic complications were less often with good or full adherence.

## 4. Discussion

Our study using real-world individual patient data revealed that treatment adherence to OACs in patients with AF following stroke was modest: the mean treatment adherence was 81%. Although the mean adherence was good, only 68% of the patients had good or full adherence during the first year after stroke. Moreover, if we take into account the correct dosage of OACs, only 50% of patients received sufficient prevention. The only factor contributing to good adherence was first-ever stroke. In majority of the patients, AF had been diagnosed before stroke, but less than half of these patients were on anticoagulants. The underuse of OACs in patients with AF prior to stroke has also been shown by other studies [4, 6–8, 14, 20].

Adherence to a medication is the mainstay of effective treatment. Poor adherence to any medication can be intentional or unintentional and can be affected by many patient-related factors [5]. While a few studies have reported excellent poststroke medication adherence [6, 8, 10], most authors have reported only a 61–77% adherence to OAC treatment [7, 9]. One large observational study from United States has also shown that hospitalisations due to thromboembolic conditions (including stroke) decrease treatment adherence to OACs in patients with AF [21].

Most previous studies with stroke patients have not reported differences in sex [6–9, 12]. We report a nonsignificant trend towards better adherence among women; similar results were found by a Swedish study [11]. The adherence was not affected by stroke severity, CHA_2_DS_2_-VASc, or the HAS-BLED scores.

We found a tendency of better adherence to apixaban but not to other OACs. A Swiss registry including stroke patients did not find a significant difference in the adherence to various OACs, but the proportion of fully adherent patients was lowest in the dabigatran group [6]. A trend towards lower adherence to dabigatran was shown also by other studies [20, 22, 23].

It is crucial to start the treatment during hospital stay if the stroke is not too severe. In our study, the treatment was started only by 65% of those who were prescribed treatment initiation at home. Similar results were reported by two recent German studies [7, 8].

We used individual patient follow-up data to assess correctness of prescribed dosages and found that in majority of the patients (66%), the prescribed reduced dose was not justified. In these cases, the patient may be adherent to treatment, but the incorrect dosage does not provide the efficacy needed.

It is difficult to compare the results with other studies because of several methodological differences. Abdou et al. recommended using different data sources to assess medication adherence to ensure more solid results [5]. Most of the previous studies on poststroke OAC adherence used the interview method (patient-reported adherence) [6–13]. This method does not assure that there has not been any treatment caps and unintentional poor adherence (for example, due to cognitive dysfunction) could be underestimated. If only prescription data is used, indicated or justified treatment gaps might be counted as poor adherence, and if only patient-reported data is used, there is a risk of underestimating poor adherence due to specific patient-related factors. The differences in the studies, cohorts, and methods used are likely one of the reasons why the results of different studies have not been consistent.

Most of the real-world studies available did not evaluate the complications of OAC treatment following stroke, and this should be taken into account when comparing the results. The rate of complications (both thromboembolic and hemorrhagic) was somewhat higher in our cohort compared to that in randomized controlled trials [1]. It is important to note that our patients already had a stroke while the randomized trials included also patients with no history of thromboembolic disease. However, the mean age of our population and the mean CHA_2_DS_2_-VASc were higher and the sample size is smaller than in the randomized trials, which could have contributed to this result. In addition, the randomized controlled trials used CHADS_2_ scoring which takes into account less risk factors and thus gives lower scores compared to the CHA_2_DS_2_-VASc. While most of the patients with hemorrhagic complications were adherent to treatment, there were still approximately half of the patients with thromboembolic events who were nonadherent.

The strength of our study was the use of real-world individual patient data from different sources, resulting in high-quality data. In this study, we used not only administrative data but also individual case histories, clinical data, and information about any hospitalizations and outpatient visits during the follow-up period that we carefully reviewed and analyzed to detect cases with complications and information about factors that could influence the treatment adherence. If we had not looked into patient files and used only administrative data, we would have overestimated the proportion of patients with sufficient prevention by 50%. In addition to adherence, we evaluated treatment complications and the validity of the prescribed dosage in order to assess both the risks and benefits associated with OACs. As all health information for each individual in Estonia is digital, we did not lose any cases during the follow-up.

One limitation of our study might have been that we measured adherence based on dispensed prescriptions, expecting that the medications were taken by the patient. This approach could have caused us to overestimate drug adherence, though we considered this possibility highly unlikely. Also, patients who were prescribed OAC but never started the treatment were excluded from the analysis. Including those patients in the nonadherent group would have further decreased the adherence rate. In addition, we did not assess the adherence to OAC treatment prior to stroke occurrence, which could have added valuable information and could have explained the causes for stroke recurrence. While it is known that OACs do not prevent all strokes even if used correctly, a relatively high proportion of patients (33%) in our cohort on prior OAC treatment suffered from stroke. An even higher proportion has been reported by the Erlangen cohort [8]. Secondly, our sample size is relatively small, but we included all consequent patients and performed a detailed analysis of individual data. Another limitation was that we did not have the data on INR measurements in patients who used warfarin; therefore, the adherence of that group was evaluated on first dosage alone.

## 5. Conclusion

Our study showed that OAC treatment adherence following stroke was modest and must be improved in order to prevent recurrent stroke. While it is generally accepted that adherence ≥ 80 % is considered sufficient, this means that 20% of the time, the patient is still at risk of thromboembolic complications. It is important to be knowledgeable of modifiable factors of treatment adherence and to manage them in everyday practice. Due to the fixed dosing and stable pharmacokinetics, NOACs do not require monitoring in principle, but at the same time, patient monitoring and long-term surveillance seem obligatory. As a future perspective, patients should be educated and reminded of the efficacy of OAC treatment for AF and the importance of treatment continuity as well as physicians should be encouraged to use OAC in patients with AF according to guidelines, to evaluate the risks and benefits of the treatment in each individual patient before discontinuing the treatment or using the reduced dosage.

## Figures and Tables

**Table 1:** Patient characteristics comparing nonadherent patients, patients with good adherence, and patients with full adherence (*N* = 202).

	Nonadherence MPR < 80%	Good adherence MPR ≥ 80–99%	Full adherence MPR ≥ 100%	*p* value
Patients, *N* (%)	66 (32%)	58 (29%)	78 (39%)	
Male, *N* (%)	28 (33%)	28 (35%)	30 (32%)	
Female, *N* (%)	38 (33%)	30 (26%)	48 (41%)	0.519
Mean age (y, SD)	76.8 (7.48)	76.0 (10.31)	76.0 (9.04)	0.201
First stroke, *N* (%)	41 (27%)	46 (30%)	66 (43%)	0.005
Recurrent stroke, *N* (%)	25 (51%)	12 (25%)	12 (24%)	
Mean CHA_2_DS_2_-VASc	5.87 (1.51)	5.46 (1.60)	5.51 (1.53)	0.248
Mean HAS-BLED	2.41 (0.72)	2.48 (0.80)	2.28 (0.79)	0.306
Mean eGFR (SD)	67.4 (19.5)	67.9 (22.1)	68.4 (18.1)	0.951
NIHSS at discharge median (IQR)	2 (1-4.5)	2 (1-4)	2 (0-5)	0.965
Rivaroxaban, *N* (%)	21 (34%)	22 (35%)	19 (31%)	0.247
Apixaban, *N* (%)	25 (30%)	19 (23%)	39 (47%)	
Dabigatran, *N* (%)	10 (34%)	10 (34%)	9 (31%)	
Warfarin, *N* (%)	10 (36%)	7 (25%)	11 (39%)	

MPR: medication possession ratio; SD: standard deviation; IQR: interquartile range; eGFR: estimated glomerular filtration rate; NIHSS: National Institutes of Health Stroke Scale.

**Table 2:** Logistic regression model for factors related to good/full treatment adherence (MPR ≥ 80%).

	Odds ratio ^†^	SE	*z*-value	*p* value	95% confidence interval
Age (y)	1.00	0.02	-0.12	0.90	0.95-1.04
Female	1.00				
Male	0.79	0.30	-0.64	0.52	0.38-1.64
Recurrent stroke	1.00				
First-ever stroke	2.27	0.82	2.26	0.02	1.12-4.61
CHA_2_DS_2_-VASc	0.79	0.11	-1.62	0.11	0.60-1.05
HAS-BLED	1.07	0.26	0.29	0.77	0.67-1.72
eGFR	1.00	0.01	0.36	0.72	0.98-1.02
NIHSS at discharge	1.00	0.04	-0.35	0.73	0.92-1.06
Apixaban	1.00				
Dabigatran	0.75	0.36	-0.60	0.55	0.29-1.94
Rivaroxaban	0.92	0.36	-0.21	0.84	0.43-1.99
Warfarin	0.63	0.31	-0.93	0.35	0.24-1.65

^†^Odds ratios are mutually adjusted for all variables presented in the table. MPR: medication possession ratio; SE: standard error; eGFR: estimated glomerular filtration rate; NIHSS: National Institutes of Health Stroke Scale.

## Data Availability

The data that support the findings of this study are available from the corresponding author upon reasonable request.

## Abbreviations

OAC: Oral anticoagulation
NOAC: Non-vitamin K antagonist oral anticoagulant
AF: Atrial fibrillation
EP: Estonian Electronic Prescription system
HER: Estonian Electronic Health Record system
NIHSS: National Institutes of Health Stroke Scale
MPR: Medication possession ratio
GFR: Glomerular filtration rate.

## Supplementary Materials

Supplementary Materials Supplementary Table 1: the patient demographics are shown.
