# Supplementary material for: Deceptive Adherence to Anticoagulation in Secondary Stroke Prevention
Source: Stroke Res Treat. 2022 Jul 11;2022:5318259. doi: 10.1155/2022/5318259 (PMC9293572; doi:10.1155/2022/5318259)
Supplement: Supplementary Materials — Supplementary Table 1: the patient demographics are shown. [file 5318259.f1.docx]

Supplementary Table 1. Patient demographics.

|  | Male | Female | Total | p-value |
| --- | --- | --- | --- | --- |
| Number of patients (%) | 134 (38) | 219 (62) | 353 (100) |  |
| Mean age, years (SD) | 74.6 (10.4) | 81.5 (7.9) | 78.8 (9.5) | <0.001 |
| CHA_2_DS_2_-VASc, median (IQR) | 5 (4-6) | 7 (6-7) | 6 (5-7) | <0.001 |
| HAS-BLED, median (IQR) | 2 (2-3) | 2 (2-3) | 2 (2-3) | 0.043 |
| AF diagnosed before index stroke (%) | 93 (69) | 158 (72) | 251 (71) | 0.581 |
| Recurrent stroke (%) | 43 (32) | 61 (28) | 104 (29) | 0.357 |
| Mean eGFR, mL/min/1.73m^2^ (SD) | 70.6 (20.3) | 62.4 (20.4) | 65.5 (20.7) | <0.001 |
| eGFR, mL/min/1.73m^2^ n (%)  <30  30-50  >50 | 2 (1)  21 (16)  110 (83) | 13 (6)  54 (24)  151 (69) | 15 (4)  75 (21)  261 (74) | 0.011 |
| OAC use prior to admission n (%) | 49 (37) | 67 (31) | 116 (33) | 0.246 |
| NIHSS (median, IQR)  On admission  At discharge | 8 (3-15)  3 (1-10) | 10 (5-18)  4 (1-13) | 9 (4-17)  4 (1-12) | 0.049  0.416 |

SD (standard deviation); IQR (interquartile range); AF (atrial fibrillation); eGFR (estimated glomerular filtration rate); OAC (oral anticoagulants); NIHSS (National Institutes of Health Stroke Scale)
